# Separate and Combined Effects of DNMT and HDAC Inhibitors in Treating Human Multi-Drug Resistant Osteosarcoma HosDXR150 Cell Line

**DOI:** 10.1371/journal.pone.0095596

**Published:** 2014-04-22

**Authors:** Enrico Capobianco, Antonio Mora, Dario La Sala, Annalisa Roberti, Nazar Zaki, Elarbi Badidi, Monia Taranta, Caterina Cinti

**Affiliations:** 1 Laboratory of Integrative Systems Medicine (LISM), Institute of Clinical Physiology, CNR, Pisa, Italy; 2 Center for Computational Science (CCS), University of Miami, Miami, Florida, United States of America; 3 College of Information Technology (CIT), United Arab Emirates University (UAEU), Al Ain, UAE; 4 Institute of Clinical Physiology, Experimental Oncology Unit, CNR, Siena, Italy; 5 Department of Experimental Biomedicine & Clinical Neuroscience, Policlinico Universitario, Palermo, Italy; 6 Ovarian Cancer Research Center, Perelman School of Medicine, University of Pennsylvania, Philadelphia, Pennsylvania, United States of America; University of Navarra, Spain

## Abstract

Understanding the molecular mechanisms underlying multi-drug resistance (MDR) is one of the major challenges in current cancer research. A phenomenon which is common to both intrinsic and acquired resistance, is the aberrant alteration of gene expression in drug-resistant cancers. Although such dysregulation depends on many possible causes, an epigenetic characterization is considered a main driver. Recent studies have suggested a direct role for epigenetic inactivation of genes in determining tumor chemo-sensitivity. We investigated the effects of the inhibition of DNA methyltransferase (DNMT) and hystone deacethylase (HDAC), considered to reverse the epigenetic aberrations and lead to the re-expression of *de novo* methylated genes in MDR osteosarcoma (OS) cells. Based on our analysis of the HosDXR150 cell line, we found that in order to reduce cell proliferation, co-treatment of MDR OS cells with DNMT (5-Aza-dC, DAC) and HDAC (Trichostatin A, TSA) inhibitors is more effective than relying on each treatment alone. In re-expressing epigenetically silenced genes induced by treatments, a very specific regulation takes place which suggests that methylation and de-acetylation have occurred either separately or simultaneously to determine MDR OS phenotype. In particular, functional relationships have been reported after measuring differential gene expression, indicating that MDR OS cells acquired growth and survival advantage by simultaneous epigenetic inactivation of both multiple p53-independent apoptotic signals and osteoblast differentiation pathways. Furthermore, co-treatment results more efficient in inducing the re-expression of some main pathways according to the computed enrichment, thus emphasizing its potential towards representing an effective therapeutic option for MDR OS.

## Introduction

OS is one of the most prevalent primary malignant bone tumors, showing high incidence in adolescence and above the age of 50 years, and representing the second leading cause of cancer-related death [1], [2]. Approximately 20% of patients present with metastasis *at the time* of initial *diagnosis*, and 50% of the rest of patients are destined to develop metastatic disease during treatment [3]. The unfavorable prognosis, and the low efficacy of chemotherapy in patients with metastasis or relapsed disease overall indicating a 5-year survival rate of <20%, makes the discovery of novel and improved therapeutic options particularly urgent [4]. While OS pathogenesis is not yet clear, substantial evidence suggests that OS should be considered a differentiation disease [5]. The rationale behind this hypothesis is that the terminal differentiation of osteoblasts derived from multipotent mesenchymal stem cells, represents a highly structured process whose control depends on a cascade of regulatory genes possibly silenced during OS development. In particular, specific OS molecular features suggest that both genetic and epigenetic disruption of osteoblast differentiation pathways may occur during tumor development [6]–[8].

Our driving hypothesis is that promoting differentiation and/or circumventing differentiation defects should be considered before undertaking effective OS adjuvant therapies. Based on the fact that genetic and epigenetic processes are synergistic drivers of malignancy, it is crucial to know the timing of such synergy. Notably, the aberrant methylation can begin very early in cancer progression, and mediate most of the important pathway abnormalities, including loss of cell cycle control, alteration of function of transcription factors and receptors, disruption of normal cell-cell and cell-substratum interaction, inactivation of signal transduction pathways, loss of apoptotic signals and genetic instability, among other possible effects.

Recently, cancer genome charts have addressed the prominent role of epigenetic regulation. In particular, a central idea is that the acquisition of additional epigenetic/genetic modifications may lead to the development of drug resistance phenotypes [7], [9], [10]. Following this idea, a few specific aspects need to be highlighted. First, epigenetic inactivation of genes can directly determine tumor chemo-sensitivity, based on known studies [11]–[14] pointing out the potential for influencing drug resistance and post-therapy clinical outcomes [15], [16]. Second, the so-called DNA methylation and histone modifications paradox deserves special attention. Both processes manifest through de-repression of oncogenes and silencing of genes involved in key DNA damage responses pathways and DNA repair during malignant transformation/progression. However, at the gene expression scale such co-activity of both DNA methylation and histone modifications can be either independent or dependent [17], being centered on silencing gene expression through modulation of transcription factors and condensation of local chromatin structure [18]. Finally, epigenetic modifications induce gene expression profiles, which vary widely across cancers, reflecting a model in which methylation and/or histone de-acetylation observed for certain genes would give a growth or survival advantage to cancer cells. Consequently, aberrant patterns of methylation would emerge depending on the selective pressure for gene silencing in each specific cancer type [19], and simultaneous inactivation of several pathways would occur, compromising cell survival or cancer progression genes [20].

The fact that epigenetic processes can be reverted, provides the rationale for using chromatin re-modeling agents, DNMT inhibitors and HDAC inhibitors, which induce the epigenetic re-programming needed for the restoration of normal expression of cancer-suppressor genes [21]. A substantial clinical impact is expected from epigenetically-driven drugs designed to change acquired drug resistance which is associated with epigenomic features, and to prevent or reverse non-responsiveness to anti-cancer drugs [16]. An important observation has been made about the selection of MDR HosDXR150 cells by culturing sensitive Hos cell lines in the presence of increasing doxorubicin doses: the clones acquire cross-resistance to chemotherapeutic drugs by overexpressing the P-glycoprotein, and by epigentically silencing TP73, thus leading to knockout of apoptotic response [14]. This observation has in turn represented the basis of a captivating model for the identification of the molecular markers and pathways contributing to OS progression and MDR phenotypic selection. Such model is empowered to test potential “epigenetic drugs” or treatment options for non-responsive OS patients. The aim of the present work is to show the effectiveness of treatments based on DNMT and HDAC inhibitors in inducing growth arrest, apoptotic response and reprogramming of MDR OS phenotype towards osteoblast differentiation. We demonstrated the influence exerted over the expression of epigenetically modified genes, and provided insights on the mechanisms by which epigenetic therapeutic options can fight metastatic and non-responsive OS.

## Materials and Methods

### Ethics Statement

Doxorubicin-resistant osteosarcoma cells (HosDXR150) origin from commercial Hos cell line (American type culture collection, ATCC, Rockville, MD). HosDXR150 cells were previously selected in our laboratory throughout a continuous exposure of Hos cells to increasing doses of doxorubicin hydrochloride (DXR). The characterization of this HosDXR150 osteosarcoma cells, has been published by us in the paper: La Sala D et al. (2003) Oncogene 22∶3518–3529.

### Cell Culture and Treatment

Doxorubicin-resistant osteosarcoma cells (HosDXR150) were obtained by continuous exposure of parental OS sensitive Hos cells (American type culture collection, ATCC, Rockville, MD) to increasing doses of doxorubicin hydrochloride (DXR) 0.5–150 ng/ml. This proprietary drug resistant HosDXR150 human OS cell line was cultured in Dulbecco’s Modified Eagle Medium (D-MEM) supplemented with 10% heat inactivated FCS at 37°C in humidified, 5% CO2 atmosphere. 2,5 uM 5-Aza-2′-deoxycytidine (5-Aza-dC), 300 nM Trichostatin A (TSA) or both in combination were added to cell culture. In the experiments where both drugs were used, the TSA was added to medium culture 12h after the 5-Aza-dC.

### Cell Viability

Quantitative cell viability was measured by colorimetric assay using cell proliferation (MTT kit) (Roche Molecular Biochemicals, Germany). 5×10^3^ cells/well HosDXR150 cells were grown in microtiter plates (96-well) in a final volume of 100 ul culture medium. The incubation period of cells culture was 24, 36, 48, 72 and 96 hours in the absence (control) or presence of drugs. 10 ul of MTT labeling reagent was added to each well at final concentration 0.5 ug/ml. MTT was cleaved by growing cells to form purple formazan crystals and allow quantification by spectrophotometric analysis (ELISA). The optical density (OD) values were measured at 550 nm on a multifunctional microplate reader (Tecan, Durham, NC, USA). Cell viability was expressed as the percentage of the ratio between the absorbance of drug-treated cells relative to that of the control (untreated) cells according to the formula: 1-experimental group OD/control group OD)×100%. Results were plotted as the mean ± SD (standard deviation) of 3 separate experiments from 6 determinations per experiment at each experimental condition.

### RNA Preparation

Total RNA samples were isolated from treated and untreated HosDXR150 cells after 48h of cell cultures using TRIZOL reagent (Invitrogen, CA, USA) according to the manufacturer’s instructions. Concentration of purified RNA samples were determined by A260 measurement and the quality was checked by Lab-on-a-chip analysis (total RNA nanobiosizing assay, Agilent) with the Agilent 2100 Bioanalyzer.

### cDNA Microarray

RNAs isolated from treated and untreated HosDXR150, and transcribed in cDNAs, were used to carry out the microarray analysis. The cDNAs hybridization was done on a microarray chip called *MWG Human Cancer Array* purchased from MWG Biotech AG. This microarray contain 50-mer oligo-probes for 1920 genes (1853 human genes associated with cancer, 27 control genes and 40 replicated genes). Microarray analysis was performed by MWG Hybridization Service (MWG Biotech AG). For each experimental point 10 ug of total RNA from a control (reference pool) and from the sample (test pool) are labeled with Cy3 and Cy5 respectively, utilizing a 2-step aminoallyl labeling. Co-hybridization with the Cy3- and Cy5-probe is performed in an hybridization station on a MWG Human Cancer Array (MWG Biotech AG). Every channel (Cy3, Cy5) is scanned three times with increasing photomultiplier gain settings using a Scanner 418/428 (Affimetrix) at 10 µm resolution ensuring coverage of the full dynamic range. The produced 16-bit tiff images are used to perform the analysis. The ImaGene pixel selection algorithms (BioDiscovery) determine signal and background intensities for every individual spot. Spots flagged as low quality were excluded from further analysis. Data is freely available to researchers upon request. The requests can be sent to Dr. Caterina Cinti, the corresponding author of this paper.

### Data Analysis

The ImaGene intensity values are processed using MAVI software (MWG Biotech AG), which solves saturation and calculates the normalization parameters. GeneSight 4.0 (BioDiscovery) is used to linearize the intensity values, calculate differential expression and perform gene-clustering analysis. Genes were selected as having at least 1.5-fold change in the log2 ratios of expression level and statistically significant (Wilcoxon test) at the 0.01 cutoff P value; Benjamini and Hochberg correction for multiple testing was applied. We used “R 3.0.1” for post-processing of the micro-array results. The selected genes were those whose log2 ratio was greater than or equal to 1.5 (up-regulated) or less than or equal to −1.5 (down-regulated).

For GO term analysis, annotation was added using the packages “org. Hs.eg.db” v.2.9.0 and “GO.db” v.2.9.0. The frequency of a GO term was defined as the number of times that the term appears in a set of genes divided by the size of the set. The difference between frequencies of two sets of genes (such as two different treatments) was used as a first indicator of the differences between those sets (a positive value points to terms enriched in the first set and not in the second one, while a negative value suggests the opposite). Venn diagrams were generated using “limma” v.3.17.23. For pathway enrichment analysis, we used the software “ClueGO 2.0.6” for “Cytoscape 3.0.1”, applying the “Function” analysis mode and the “Compare” cluster analysis type (cluster 1 = up-regulated genes and cluster 2 = down-regulated genes) for each of the treatments (DAC, TSA and combined DAC+TSA). The statistical test used for enrichment in all cases was the right-sided hypergeometric test. We selected only the terms with a p-value smaller than 0.05 and at least three genes per term, with a multiple testing correction using the Benjamini-Hochberg method. The pathway databases included KEGG, REACTOME, WikiPathways and the Biological Process Gene Ontology, updated to 10.09.2013. The GO tree levels to use were set to a minimum of 5 and maximum 20. We fixed the value of the kappa estimator of edge significance to 0.5. The other parameters of the software were set to default values (for example, the “GO term fusion” option was not activated).

### Quantitative Real Time- PCR (qRT-PCR)

Total RNA was extracted from treated and untreated HosDXR150 cells using TRIZOL (Invitrogen, CA, USA) according to the manufacturer’s instructions. For each sample, 2 mg of total RNA previously used for microarray analysis was reversely transcribed using the QuantiTect Reverse Transcription Kit (Qiagen Inc., Valencia, CA) according to the manufacturer’s instructions. Gene expression was determined using the DyNAmo Flash SYBR Green qPCR Kit (Thermo Scientific Inc., USA) on the PikoReal Real-Time PCR System (Thermo Scientific Inc., USA). *Primers were* as follows:

- CASP10 sense 5′-TCCCAAGCAAATGGGAGCTTCT-3′, reverse 5′-TCATGGCCAGCCTTCAGATCAA-3′;
- IL12A sense 5′-ACCTCAGTTTGGCCAGAAACCT-3′, reverse 5′-AGAGTTTGTCTGGCCTTCTGGA-3′;
- REL A sense 5′-TTGAGGTGTATTTCACGGGACC-3′, reverse 5′-ACTGCACCAGTGAGATCAGGA-3′;
- TP73 sense 5′-GACGAGGACACGTACTACCTT-3′, reverse 5′-CTGCCGATAGGAGTCCACCA-3′;
- TP53 sense 5′-ACGGAACAGCTTTGAGGTGCAT-3′, reverse 5′-TGTTGGACAGTGCTCGCTTAGT-3′;
- TNFRSF6 sense 5′-AAAGCTAGGGACTGCACAGTCA-3′, reverse 5′-GTCCGGGTGCAGTTTATTTCCA-3′;
- RBL2 sense 5′-ATGCTGTCCCTGTGCAGAATGT-3′, reverse 5′-CTTGCACAGGAATGGTTACCGT-3′;
- VEGFA sense 5′-AGCTACTGCCATCCAATCGAGA-3′, reverse 5′-TGCATGGTGATGTTGGACTCCT-3′;
- ACTIN sense 5′-TGCGTGACATTAAGGAGAAG-3′, reverse 5′-GCTCGTAGCTCTTCTCCA-3′.

Amplification conditions were: 7 minutes at 95°C, followed by 40 cycles of 10 seconds at 95°C, 20 seconds at 60°C and 20 seconds at 72°C. All assays were carried out in triplicate. The relative expression of target genes was evaluated using the comparative cycle threshold method, with b-actin used for normalization.

### Western Blotting Analysis

Treated and untreated HosDXR150 cells were lysed in 50 mM Tris/HCl, 5 mM EDTA, 250 mM NaCl, 50 mM NaF, 0.1% Triton-X, 0.1 mM Na_3_VO_4_ plus protease inhibitors (Roche Molecular Biochemicals, Germany). Equal amounts of proteins were resolved on a 7% or 10% SDS-PAGE, transferred onto nitrocellulose membranes, and then successively blocked with 5% non-fat dry milk. Anti-RBL2 was from BD Transduction Laboratories. Anti-FAS, anti-CASP10 and anti-IL12A were from Sigma-Aldrich. Anti-TP73, anti-TP53, anti-actin and the horseradish peroxidase secondary antibodies were from Santa Cruz Biotechnology. Primary antibodies were used at a dilution of 1∶200. Secondary antibodies were used at a dilution of 1∶5000. Signals were acquired using the ImageQuant LAS 4000 (GE Healthcare).

## Results

We investigated the epigenetic mechanisms occurring in the MDR OS phenotype by treating HosDXR150 cells with two agents, first separately and then in combination:

- A de-methylating agent alone, i.e. 5-Aza-dC (or DAC), an inhibitor of DNMT;
- An inhibitor of histone de-acetylases (HDAC), i.e. the Tricostatin A (TSA);
- A combination of i) and ii).

**Figure 1:**
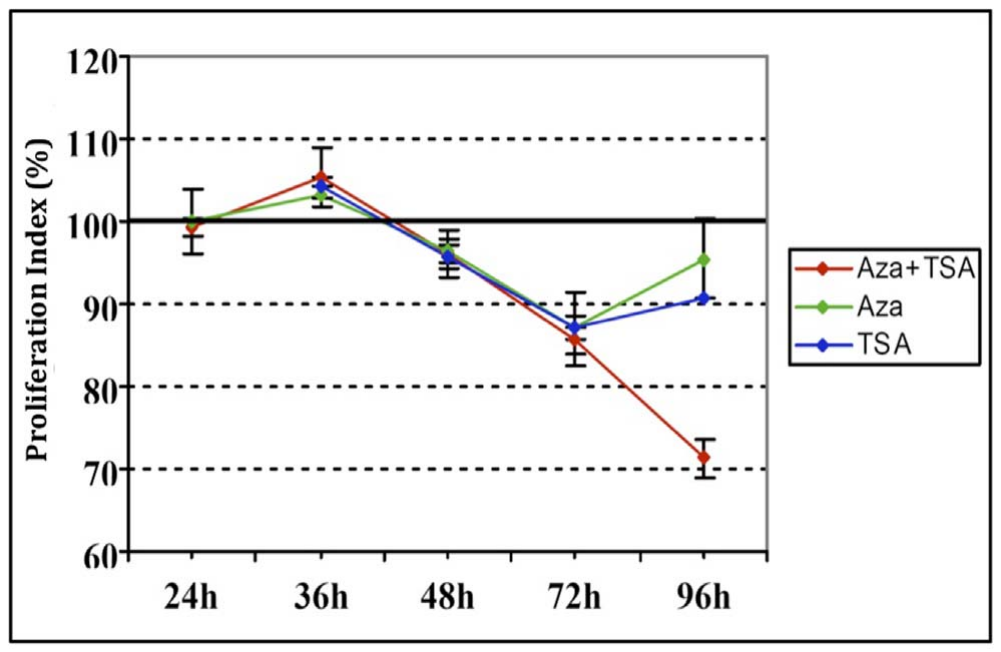
Anti-proliferative effects of 5-Aza-dC and/or TSA in MDR osteosarcoma (Hos DXR150) cell line after 24, 36, 48, 72, 96 hours from treatment. The percentage of proliferation index +/− the SD has been reported in the y axis. The mean ± SD of 3 separate experiments from 6 determinations per experiment at each experimental condition. AZA (green line): cells treated with 5-Aza-2′-deoxcycitudine (DAC); TSA (bleu line): cells treated with Tricostatin A; AZA+TSA (red line): cells treated with both drugs (DAC+TSA).

Figure 1 reports the anti-proliferative effects of the agents described in i), ii) and iii). The cell viability during time exposure to drug treatments was assessed by colorimetric assay using a cell proliferation kit (MTT). While the single treatments (green line for 5-Aza-dC and blue line for TSA) have a limited effect in reducing cell viability with time, up to 72 hours, the co-treatment induces constant inhibition on cell proliferation at all times of treatment, suggesting increased efficacy in reducing proliferation of MDR OS cells compared to each of the individual treatments.

DNMT and HDAC inhibitors have a distinct mechanism of action in determining the re-expression of epigenetically modified genes. Therefore, the identification of genes with expression modulated by the treatments is the first of our desired steps designed to assess whether the agents may remove the transcriptional repression of genes, and re-activate the apoptotic response in MDR OS cells. In order to establish the effect of each treatment, we performed cDNA microarray and functional analyses targeting genes whose expression is drug-modulated separately as well as jointly.

**Figure 2:**
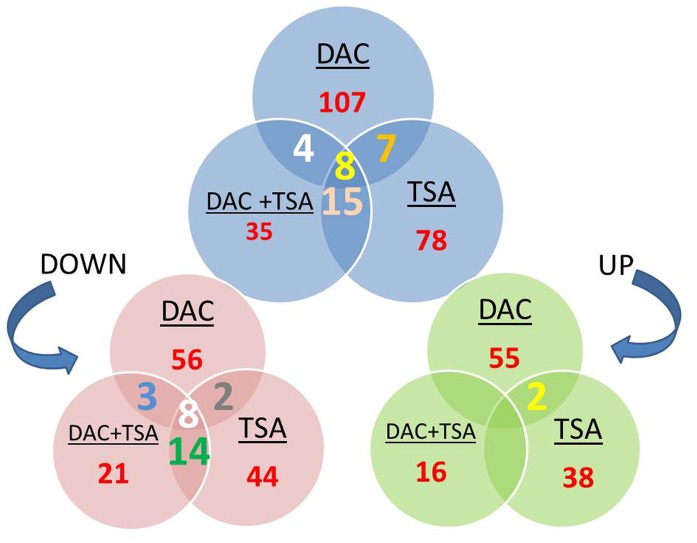
Venn diagrams representing overlap between differentially expressed genes after each treatment. (a) Overlap between all DE genes after treatments with DAC, TSA and DAC+TSA in MDR OS; (b) Overlap between up-regulated genes after treatments with DAC, TSA and DAC+TSA; (c) Overlap between down-regulated genes after treatment with DAC, TSA and DAC+TSA.

Table 1 summarizes the differentially expressed (DE) genes after each treatment. The gene lists obtained from each condition were divided into three groups by *k-means* clustering. The type of regulation (up or down) for each of the resulting 9 clusters is specified in the last column of Table 1. Venn diagrams indicating the genes regulated after the three conditions are in Figure 2. While up-regulated genes seem to be very specific to each treatment, several down-regulated genes are present in both separate and combined treatments. Both treatment-specific and shared pathways linked to the genes listed in Figure 2 are provided by functional enrichment. The specifications of the used software, *ClueGO* [22], are described in Materials and Methods. Based on the outcomes of such tool, Figures (Ss) S1, S2, and S3 show functionally enriched clusters based on up- and down-regulated genes. Tables (S1–8) report GO terms and corrected p-values for all genes associated to each annotated functional cluster.

**Table 1:** Up and down-regulated genes after each treatment (DAC, TSA, combination).

Cluster	N. ofGenes	Genes	Regulation
**DAC –1**	**(69)**	*CDH17, C14orf118, ST5, BRIT1, SEMA3B, SAFB, DAG1, RELA, SOTV, BRDT, GUK1, GSTA4, UBL3, TCF12, BCAN, GTPBP1, USP32P1, MYBL1, TNFRSF18, VIPR1, TFPI2, T3G, OPRD1, DBCCR1, SCYA8, ISLR, PCTK1, MYCL2, TSG101, PSG1, CDH6, FKSG2, VAULT1, RAB27B, ENG, HSD17B3, ACVRL1, CTSO, SLC1A5, CEACAM5, NY-REN-60, MCC, CFTR, SIAT8B, FMR2P, BAI1, SLC29A2, MAPK1, MYC, MAGEA12, NPD002, MMP2, LY6E, FGF20, STAG1, SSX3, FES, CD8B1, IFNB1, PGF, TFE3, PTGES, SCYB13, TNFRSF7, DAP5, MADH5, MGB2, EI24, PSG3*	**Down**
**DAC –2**	**(20)**	*H4FM, BNIP3L, CD47, ALDH9, CEP110, LYZ, TGFA, ITGA3, ABCC4, MFI2, CP, INS, MLLT3, MLLT4, SH2B2, ZNF670, PARM1, FOXO1A, FASN, XDH*	**Up**
**DAC –3**	**(38)**	*IL6ST, TP73, NR2C2, TIMP4, CDK7, PSG, TNFRSF1B, PDCD6, IL6, ANXA7, MFGE8, LHX1, HOXC6, NEF3, TOP3B, IGF1, GGH, PBX3, WNT1, CTNND1, SLC19A1, TNFSF18, RBL2, TP53BPL, CPN2, RAD50, ELL2, HTATIP2, ATP6B1, LMOD1, ASNA1, HRMT1L2, SFN, MYB, NCOA6, RAB6, TNFSF7, KLK10*	**Up**
**TSA –1**	**(9)**	*RELA, CD38, VEGF, CDKN2B, FKSG2, ROS1, GML, STAG1, MGB2*	**Down**
**TSA –2**	**(40)**	*LCK, XYLB, BLM, MSH4, RAB13, XPA, RAD17, PDCD6, SPINK1, TGFA, FAT1, ERCC6, LCN2, HD, EVI2B, PLCB3, MALT1, S100A2, ADAMTS6, RIPK3, CASP10, HEM1, CTAG1, TAX1BP1, USP4, TP53, YF13H12, CXCR4, SDCCAG43, CYP1B1, HRB, CFLAR, CRABP1, MCC, RAB1, BMP7, FAPA, MARK3, UBE1L, TRPM1*	**Up**
**TSA –3**	**(59)**	*ACTC, ALK, ST5, CTSL2, RAG1, MAGEB3, F9, CALCR, LCP1, EBI2, MARS, BCL9, TIE, ABCC4, RFXAP, FOLR1, CDH11, HHCM, USP32P1, PDGFRL, SSX1, IGFBP1, BECN1, SSTR2, INHA, MLLT3, IGF1R, GNA13, DYRK1, ARCN1, PSG1, WWOX, BRAF, LIFR, MUC13, MGAT5, ADRB2, MAGEB1, NAPSA, CRYBA1, MGB1, CBFA2T2, TNFSF15, BAI1, HGF, UC28, IMPG1, CBLB, SLC26A2, RAB23, LECT2, LHCGR, ADAM17, MTAP, FGF20, NEO1, TRB, FGF12, PSG3*	**Down**
**DAC+TSA –1**	**(16)**	*TNFRSF6, COL1A2, S100A4, XPC, FOXM1, FHL2, GTPBP1, GAGE7, LENG4, MS4A12, NDN, SLC22A1L, DPAGT1, S100A3, AXL, IL12A*	**Up**
**DAC+TSA –2**	**(4)**	*FKSG2, CBFA2T2, GML, STAG1*	**Down**
**DAC+TSA –3**	**(42)**	*ACTC, ST5, FCGR1A, CBLC, POU2AF1, TNFSF4, CD38, VEGF, FOLR1, FOLH1, CDKN2B, HHCM, USP32P1, CASP10, BIN2, SSTR2, LIG3, PSG1, STX6, CDH6, HFE, PLOD2, ZNF45, SERPINA2, STEAP, CHP2, IFNA1, ETV1, NY-REN-60, SH3BP2, BAI1, UC28, STMN2, P2RX1, CBLB, SLC26A2, ADAM17, REG3A, FGF12, MADH5, MGB2, PSG3*	**Down**

Treatment groups indicated by ‘Cluster’ obtained by k-means clustering.

Associated gene number and list in second and third columns, respectively.

The type of regulation is specified in the last column.

A summary is provided below to elucidate the main findings:

1. **DCA treatment.** **57** genes were found significantly **up-regulated**, and **69** genes were found significantly **down-regulated**. Up-regulated genes such as TP73, RBL2, CD47, TNF-1B, SFN, MYC, CDK7, RAD50, WNT1 and IL-6-ST identified GO terms listed as *apoptosis, cytochrome C release, adipogenesis* and *regulation of fat cell differentiation, cellular ion homeostasis, regulation of embryonic and dorsal spin cord development* (Table S1 and Figure S1a). Down-regulated genes such as MMP2, MAPK1, TBFRS7, REL A, SAFB, FES identified GO terms listed as *TGF-beta signaling, angiogenesis, IL-4 signaling, cartilage development* and cancer-related pathways. Furthermore, clusters identified from other down-regulated genes include *negative regulation of cell migration,* through ACVRL1, CXCL13 and MCC genes, and *negative regulation of endothelial cell proliferation,* through ACVRL1, ENG and XDH genes (Table S2 and Figure S1b). Table S3 and Figure S1c report functionally enriched terms from both up-regulated genes (IGF1, SFN and TP73) and down-regulated genes (BAI1 and EI24), and refer to the p53 signaling pathway.
2. **TSA treatment.** **40 genes** were found significantly **up-regulated**, and for two of them, PDCD6 and TGFA, similar up-regulation was observed after DAC. Then, **68 genes** were found significantly **down-regulated**. The up-regulated genes identified GO terms listed as *response to X-ray* (BLM, ERCC6, TP53), *positive regulation of apoptosis signaling* (AGFG1, LCK, TP53) *negative regulation of DNA replication* and *DNA metabolic processes* (BLM, RAD17, TP53), as well as *regulation of reactive oxygen species metabolic process involved in bone development* (BMP7) (Table S4 and Figure S2a). The down-regulated genes identified GO terms as *melanoma, ERK1 and ERK2 cascade, myotube differentiation, enriched endochondrial ossification* (BMP7, CTSV, IGF1R and VEGFA), *negative regulation of bone resorption (calcium loss)* (CD38 and VEGFA), *negative regulation of FGFR signaling* (BRAF and FGF20), *renal carcinoma* and other (Table S5 and Figure S2b). Table S6 and Figure S2c report functionally enriched terms including both up- and down-regulated genes, identifying GO terms such as *negative regulation of extrinsic apoptotic signaling pathway* (up-regulated: AGFG1, CFLAR and HTT; down-regulated: IGFBP1, RELA), *B cell differentiation* (up-regulated: ADAM17, MALT1, NCKAP1L, TP53; down-regulated: GPR183, INHA, RAG1) and *pancreatic cancer* (up-regulated: TGFA, TP53; down-regulated: BRAF, RELA, VEGFA).
3. **DAC-TSA treatment.** **16 genes** were found significantly **up-regulated**, and **46 genes** were found significantly **down-regulated**. Some of the DE genes were previously found after either DAC or TSA treatments. Regarding the 16 up-regulated genes, S100A3, AXL, IL12A, XPC and FAS identified GO terms such as *positive regulation of natural killer cell activation*, *response to UV-B*, *African trypanosomiasis*, *allograft rejection* and *Type I diabetes mellitus* (Table S7 and Figure S3a). The last three terms share the FAS and IL12A genes, which have a role in *extrinsic apoptotic pathway*. Among the down-regulated genes, 3 were also found in relation to DAC treatment (CDH6, SMAD5, USP32), and 14 in relation to TSA treatment (CDKN2B, HHCM, SLC26A2, FGF12, FOLR1, GML, PBOV1, SSTR2, ADAM17, ACTC1, VEGFA, CBLB, CBFA2T2, CD38), while 8 genes were common to all three treatments (STAG1, USP32P1, SCGB2A1, PSG1, PSG3, BAI1, FKSG2, ST5). The down-regulated genes after DAC-TSA treatment identified GO terms such as *extrinsic pathway for apoptosis* and *death receptor signaling* (ADAM17, CASP10 and FAS), *T cell mediated immunity* (HFE, IL12A and TNFSF4), as well as *T-helper 1 type immune response* (TNFSF4 and VEGFA), *negative regulation of bone resorption*, *negative regulation of bone and tissue remodeling* (CD38 and VEGFA), *positive regulation of transforming growth factor (TGF) beta receptor signaling* (ADAM17 and CDKN2B), *megakaryocyte differentiation* (CDKN2B and PSG1). These details are reported in Table S8 and in Figure S3b.

**Figure 3:**
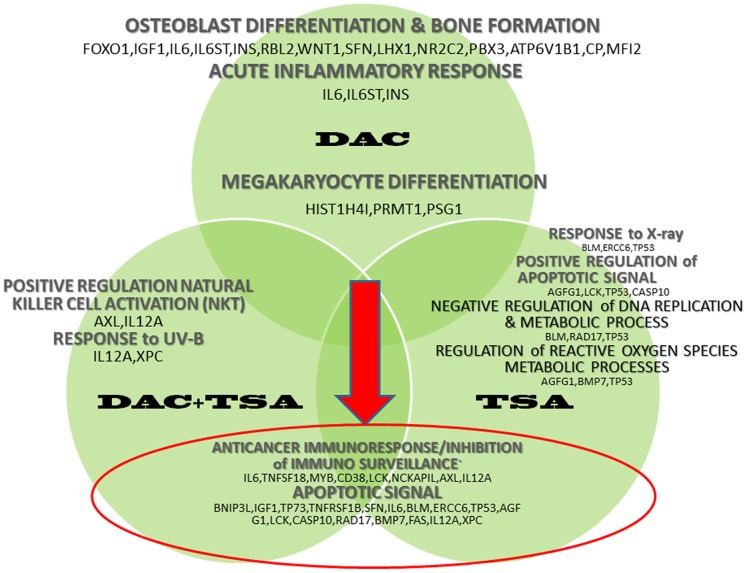
Pathways landscape. Summary of functional enrichment results showing the main pathways separated by treatment, and shared across treatments, with corresponding lists of up-regulated genes.

**Figure 4:**
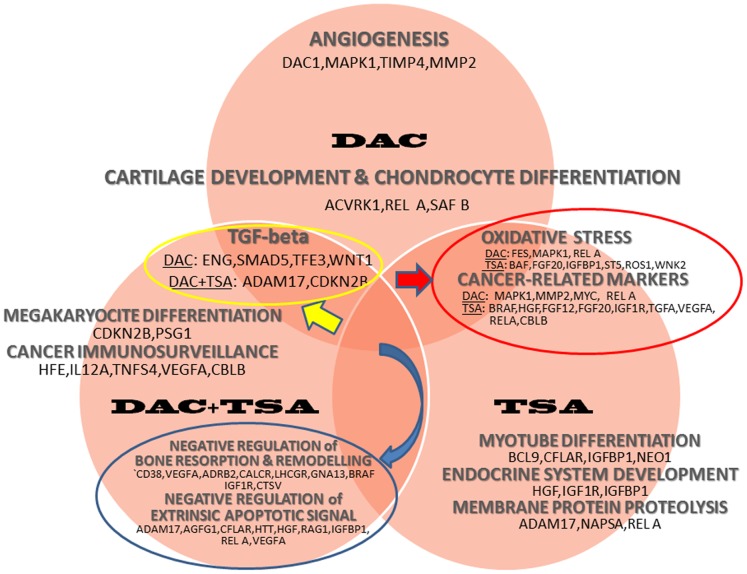
Pathway landscape. Summary of functional enrichment results showing the main pathways separated by treatment, and shared across treatments, with corresponding lists of down-regulated genes.

Figure 3 and Figure 4 report, respectively, the main up- and down-regulated pathway landscapes, together with the identified gene lists which were significantly detected after the various treatments.

**Figure 5:**
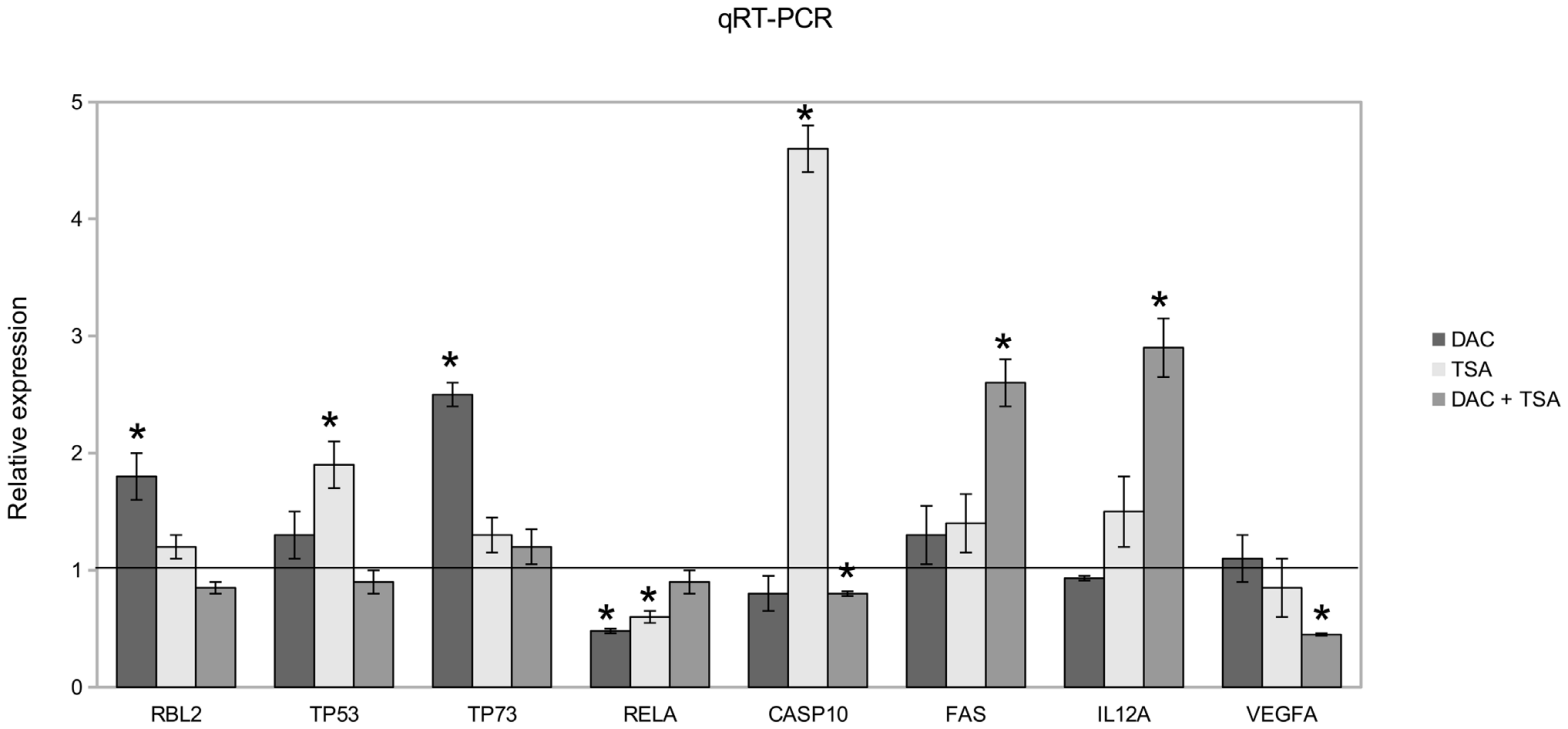
Quantitative RT-PCR analysis. The mRNA levels of selected genes were evaluated in all treated cells and compared to control. Data are normalized to β-actin. The relative expression is given as the ratio between treated and untreated cells and reported as mean ± SD. The black line represents normalized control values (*P<0.05 by Student’s t test).

**Figure 6:**
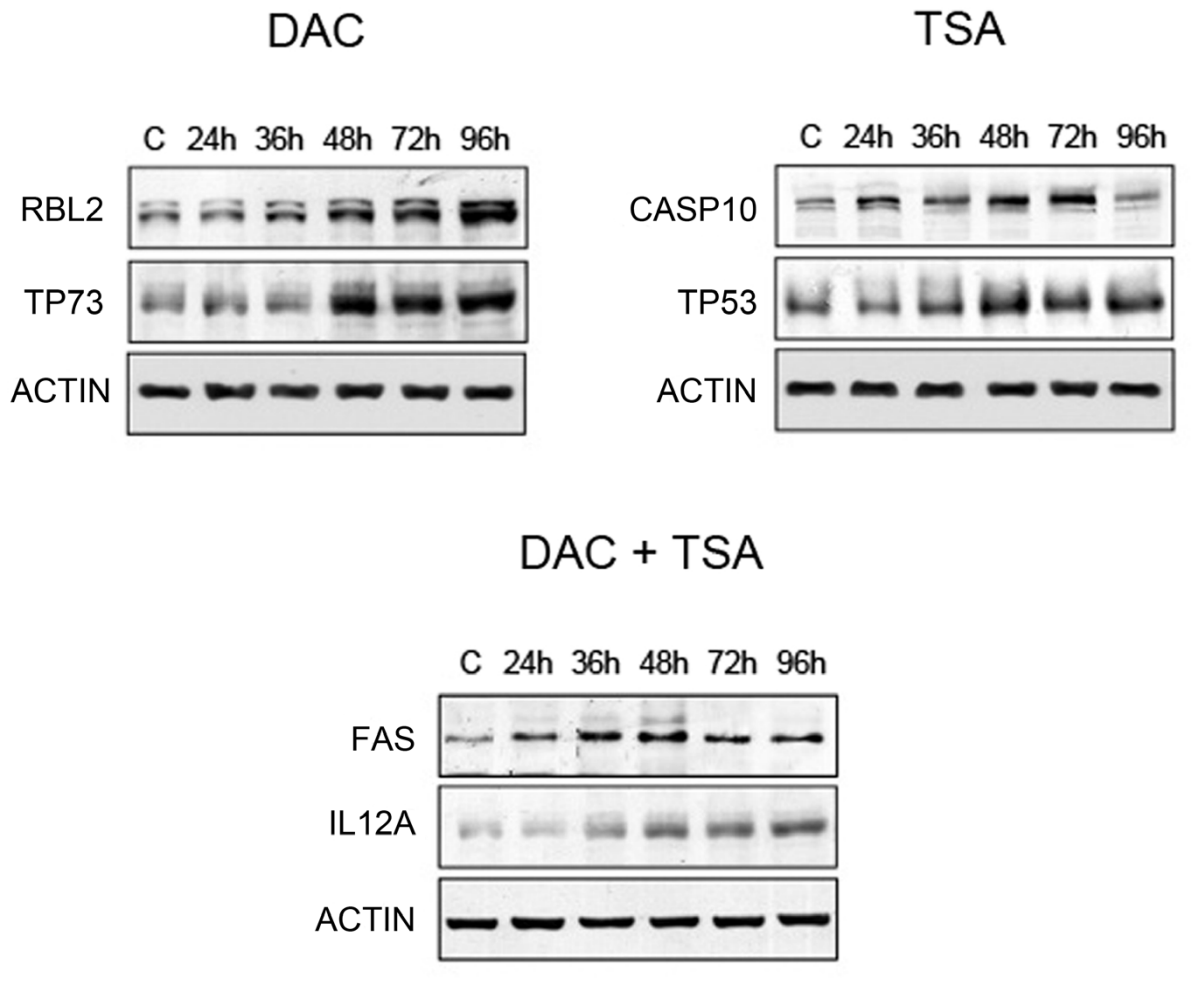
Western Blotting analysis. The expression of some proteins measured at different treatment times, and involved in apoptotic signaling and/or cell cycle control, regulated by specific treatments. A total of 40 mg of whole cell lysate from HosDXR150 treated with 5-Aza-2-deoxicytidine (DAC) or Trychostatin A (TSA) or combined treatment (DAC+TSA) at different times (24, 36, 48, 72 and 96 hours) was electrophoretically fractioned in SDS-PAGE. The levels of protein expression increased beginning at 36 hours for RBL2, CASP10 and FAS, or at 48 hours for TP73, TP53 and IL12A, and reached a maximum at 96 hours. Beta-actin was used to normalize.

Validation of 8 differentially expressed genes was performed by *qRT-PCR*; in particular, TP73, RBL2, TP53, CASP10, IL12A and FAS are selected among the up-regulated genes after specific treatment, while REL A and VEGFA are down-regulated after DAC and TSA, or TSA and DAC+TSA treatments. In Figure 5, RBL2 and TP73 appear over-expressed after treatment with DAC but not after treatment with TSA and DAC+TSA, while TP53 and CASP10 are up-regulated by treatment with TSA, and IL12A and FAS are up-regulated by treatment with DAC+TAS. REL A and VEGFA are down-regulated: the former after treatment with DAC or TSA, the latter after treatment with TSA or DAC+TSA, thus confirming the cDNA microarray analysis. Up–regulated genes were also validated by Western blotting (Figure 6), showing protein expression time course increase, and following specific treatment.

## Discussion

Despite extensive clinical trials and effective treatment regimens or therapy plans based on surgery, radiation and systemic chemotherapy, have determined improved clinical outcomes for OS patients with either relapsed or developed metastasis the therapeutic benefit of chemotherapy remains limited, suggesting that new treatment options are needed. OS appears as a malignant mesenchymal neoplasm characterized by defects in differentiation of primitive osteoblastic cells, leading to high incidence of metastasis and chemoresistance and poor prognosis [23]. In particular, chemotherapy targets mainly proliferation cells, without pinpointing potential differentiation defects of OS. Therefore, a better understanding of the molecular pathogenesis of human OS is required to be able to develop diagnostic and prognostic markers, as well as targeted therapies for patients. Some of these markers have been recently characterized in omics terms, i.e. through multi-profile analyses, supporting in turn the identification of new treatment options and the design of effective personalized therapies, especially for those patients less responsive to conventional chemotherapy [16].

Cancer stems from a cell clone with accumulated genetic and epigenetic changes that influence its phenotype, and finally enable its escape from the normal controls of proliferation [24]. Recently, cancer genomes charts have increasingly addressed the central role of epigenetic regulators in cancer, which may result in the acquisition of additional epigenetic modifications leading to drug resistance. Epigenomes may lead to differences in intrinsic sensitivity of cancer to chemotherapy, depending on the specific function of the inactivated genes, and may promote survival in the presence of drugs by allowing the selection of drug-resistant tumor cells [10], [15].

The synergy between DNA methylation and histone post-translational modifications can begin very early in cancer progression, and the profiles of gene promoter hypermethylation differ in each cancer type [19]. Several DNMT and HDAC inhibitors have been proposed to reverse the epigenetic aberrations, and induce the re-expression of all *de novo* methylated genes. In turn, this action could induce the reprogramming of cancer cells toward differentiated cells or chemo-sensitivity of MDR cancer cells [16], [21]. OS may be caused by genetic and epigenetic alterations representing a cross-sectional endpoint leading to the disruption of osteoclast differentiation pathways from mesenchymal stem cells [6], [25]. Therefore, the identification of critical epigenetic defects, which allow the OS tumors to escape from the osteoblast differentiation and apoptotic response, may lead to the design of effective epigenetic therapeutic strategies.

The applications in literature of hierarchical clustering to DNA copy number profiles of several OS cell lines and clinical samples have revealed that the difference in quality of results between cell lines and patient samples is not systematic [8]. Thus, OS cell lines can be valuable tools to study the molecular mechanisms at the basis of OS development and progression. In this work, we used the MDR OS cells, HosDXR150, obtained by continuous exposure of the parental OS sensitive cell line to increasing doses of doxorubicin, as a model to evaluate the effect of epigenetic drugs in inducing reprogramming and reverting of chemo-resistance phenotype. Previously, La Sala et al. [14] showed that an OS cell line (Hos), even with a functionally inactivated TP53 and RB1/TP105 homozygous deletion, undergoes apoptosis upon doxorubicin treatment through E2F1/TP73-dependent pathway. The p73-dependent apoptotic response is no longer functional in the MDR and metastatic variant HosDXR150 cells due to the presence of DNMT and the replacement of histone acetyl-transferase enzyme (HAT/p300) with HDAC1 on the TP73 promoter. This mechanism, in turn, triggers the DNA methylation and de-acetylation of histone tails, and consequently the epigenetic silencing of TP73 in MDR HosDXR150 cells, suggesting that further epigenetic alterations occur to clonally select the MDR OS phenotype. These observations have important clinical consequences as epigenetic modifications provide the rationale for epigenetic drugs to change reversible drug resistance-associated epigenomes, and prevent or reverse non-responsiveness OS to chemotherapeutic drugs.

We showed that DAC and TSA treatments and co-treatment induce the up-regulation of many genes, and that the co-treatment of MDR OS cells with both agents result more effective in reducing cell proliferation than any of the two component treatments, even if positive regulation of both intrinsic and extrinsic apoptotic signals seem to be the most important effect after all such treatments. However, the nature of this regulation appears highly complex, with a number of pathways being enriched in up- or down-regulated genes for all treatments, and some for each specific treatment. As a result of our multiple analyses, lesser genes appear up- and down- regulated in the combined treatment, suggesting that the effects are not simply additive but interactive, and in a quite complex way, as confirmed by functional enrichment analysis. GO and functional enrichment analyses of the microarray data, which were performed to identify groups of differentially expressed genes after all treatments, allowed us to identify several pathways and regulatory processes that might explain the experimentally observed effects.

The evidence from treatment-dependent complex regulatory processes suggests a few remarks. First of all, our data show that in the re-expression of epigenetic silenced genes (up-regulated genes), a very specific regulation takes place involving either methylation or de-acetylation, or even the two combined mechanisms. Considering the up-regulated genes reported in Venn analysis, 58 genes are exclusively re-expressed after DAC treatment, 40 genes after TSA and only 16 genes after both treatments. The differential expression profile of up-regulated genes described in this protocol seems to be mutually exclusive, and suggests that epigenetic modifications such as methylation and de-acetylation have occurred either independently or simultaneously to determine gene silencing in MDR OS. In particular, we observed that the re-activation of apoptotic response in MDR OS cells can be driven by TP73-dependent apoptotic signal in cells treated with DAC, previously described as silenced [14], and by over-expression of other pro-apoptotic genes such as AGFG1, LCK, BLM, ERCC6, AXL, XCP, FAS, CASP10 and IL12A, mainly involved in extrinsic apoptotic signaling in cells treated with TSA or DAC plus TSA. Interestingly, only the combined treatment is able to induce re-expression of IL12A, which has a role in the activation of *extrinsic apoptotic pathway* together with FAS and CASP10. However, it has also shown to have a prominent role in inflammatory responses, as well as remarkable antitumor properties by inducing immune response and inhibiting metastatic potential of OS cells via a mechanism involving the FAS/FAS ligand pathway [26], [27].

Other genes such as IL6, IL6ST, BMP7, ATP6B1, IGF1, WNT1, TNFs and ALPL, previously described as down-regulated in OS and indicated as driver genes in regulating osteoblast differentiation, adipogenesis, skeletal and bone development [6], [25], [28], result epigenetically modified in our experimental setting and are mainly up-regulated following the DAC treatment. Instead, TSA and co-treatment seem to be more effective in inhibiting negative regulators of endochondral ossification, and bone resorption and remodelling pathways via ACVR1, FGFRs, CDH11, ADRB2, CD38 and VEGFA. Interestingly, the treatments also induce the down-regulation of MMP2 and HGF genes, which have been shown over-expressed in OS patients with poor clinical outcome, and manly contribute to aggressive OS behavior, and the up-regulation of S100A6 and CXCR4, two genes described as inhibitors of metastasis.

Especially with regard to the down-regulated genes, MMP2’s expression is correlated with poor prognosis and with the ability of cell to metastatize [29]; this gene is down-regulated following the DAC treatment. Instead, for HGF, a cytokine that stimulates cell proliferation and mobility [30], a down-regulation is observed after the TSA treatment. On the contrary, S100A6, which is described to play a role in inhibition of cell mobility and anchorage-independent growth [31], is up-regulated following co-treatment, and CXCR4, which plays a role in cytoskeleton rearrangement, adhesion to endothelial cells, and directional migration [32], is up-regulated after the TSA treatment.

Other important pathways manly related to angiogenesis, oxidative stress like IL4 and ERK1/2 signaling, cell migration and proliferation like TGF-beta and cancer immunosurveillance signaling were down-regulated after the treatments. In particular, the DAC treatment seems to be more effective in inhibiting angiogenesis, cell migration/proliferation and oxidative stress through TGF-beta and IL-4 signaling respectively, while TSA seems more efficient in inducing the repression of cancer immunosurveillance beyond bone resorption and remodeling signaling. Finally, all the mentioned pathways that are down-regulated by the co-treatment suggest that the effects of each drug are additive, even if cancer immune-surveillance is silenced in addition to the above mentioned signals. The suppression of immune-surveillance may be a mechanism by which tumors resist to immune detection and elimination, and natural killer T cells play an immunoregulatory role in the biology of the OS immune-surveillance [33].

The overall of these results strongly indicates that MDR OS cells acquired growth and survival advantage by epigenetic inactivation of both multiple apoptotic signals and osteoblast differentiation pathways and by overexpression of genes involved in angiogenesis and cancer immunosurveillance signaling pathways, major related to cancer progression and MDR phenotype.

Particular gene-specific epigenetic profile acted to silence the tumor-suppressor genes and, among the three protocols of treatment, the co-treatment seems to be a more efficient therapeutic strategy in inducing tumor cell growth arrest and the reprogramming of MDR OS phenotype toward osteoblast differentiation.

Kresse et al. [8] analysed genes differentially expressed on OS cell lines and primary tumors, compared to normal bone cells, and generated a dataset of genes including those significantly methylated in their chemo-sensitive OS samples. Just 13 genes (3.3%) of the total genes described in literature as methylated genes are found differentially expressed in their study. A comparison between this set of methylated genes with the genes which we found up-regulated in the MDR OS cells after the treatments (DAC or TAS, or combined), revealed that 6 of 13 genes described in Kresse’s dataset as methylated in OS (CRABP1, RIPK3, SLC22A18, TNFRSF1B, TP73 and WNK2) were up-regulated in the MDR OS cells after one of the treatments. In contrast to their description of no effects of DAC treatment in reactivating the expression of these epigenetic silenced genes in OS cells, we found that TNFRSF1B and TP73 genes can be up-regulated by DAC treatment, that CRABP1, RIPK3 and WNK2 are up-regulated by TSA treatment, and SLC22A18 by the co-treatment.

In summary, our results indicate the following as the main findings:

1. The mechanisms by which the listed genes are silenced can refer to different epigenetic modifications. Consequently, different epigenetic treatments are needed to induce cancer cell re-programming in MDR OS;
2. The 6 genes described by Kresse et al. [8] could be considered the first events in tumor development, while the other genes that were found differentially expressed only in our dataset, can be silenced during cancer progression and MDR transformation;
3. The suggested epigenetic therapy could represent an effective adjuvant therapy for MDR OS, allowing to promote and circumvent differentiation defects of primitive osteoblast cells, and to sensitize them for a subsequent chemotherapy approach.

As a final remark, the follow-up of this study will involve a deeper investigation of regulatory mechanisms, utilization of other OS cell lines, and extension of the analysis to clinical samples.

## Conclusions

OS is the most frequent malignant primary bone tumor and represents a main cause of cancer-related death in children and adolescents. The currently available conventional therapy, which consists of multi-agent surgery, chemotherapy, and radiation, is unfortunately not totally adequate for OS treatment. Innovative drugs and treatment approaches are needed to aim at further improvements of the outcomes observed in patients. This study shows that the epigenetic silencing of most cancer suppressor and osteoblast differentiation genes, yields clonal selection of the MDR phenotype. Treatments involving both DNMT and HDAC inhibitors can induce cell growth arrest and the reprogramming of MDR-OS cells towards osteoblast differentiation. We believe that this study offers through expression experiments, analysis and annotations an understanding of mechanisms by which epigenetic therapeutic options may be useful to fight metastatic and non-responsive OS. As a final remark, we foresee in the follow-up a few novel directions of research to be pursued, for example a deeper investigation of regulatory mechanisms, the utilization of other OS cell lines, and the extension of the analysis to clinical samples.

## Supporting Information

Figure S1 Functionally enriched terms for the up- and down-regulated genes after DAC treatment. (a) Pathways and GO terms enriched in up-regulated genes after DAC treatment; (b) Pathways and GO terms enriched in down-regulated genes after DAC treatment; (c) Pathways and GO terms enriched in both up- and down-regulated genes. *ClueGO* has provided the functional clusters; the number of associated genes for each cluster are reported within the bars.
(TIF)

Figure S2 Functionally enriched terms after TSA treatment. (a) Pathways and GO terms enriched in up-regulated genes after TSA; (b) Pathways and GO terms enriched in down-regulated genes after TSA; (c) Pathways and GO terms enriched in both up- and down-regulated genes. *ClueGO* has provided the functional clusters. The number of associated genes for each cluster are reported within the bars.
(TIF)

Figure S3 Functionally enriched terms after combined DAC+TSA treatment. (a) Pathways and GO terms enriched in up-regulated genes after DAC+TSA; (b) Pathways and GO terms enriched in down-regulated genes after DAC+TSA. *ClueGO* has provided the functional clusters. The number of associated genes for each cluster are reported within the bars.
(TIF)

Table S1 Functionally enriched terms for the up-regulated genes after DAC treatment. TermIDs as from GO (Gene Ontology); WP corresponds to WikiPathways, used with KEGG and REACTOME as database sources.
(DOCX)

Table S2 Functionally enriched terms for the down-regulated genes after DAC treatment. TermIDs as from GO (Gene Ontology); WP corresponds to WikiPathways, used with KEGG and REACTOME as database sources.
(DOCX)

Table S3 Functionally enriched terms including both up- and down-regulated genes after DAC treatment. TermIDs as from GO (Gene Ontology); WP corresponds to WikiPathways, used with KEGG and REACTOME as database sources.
(DOCX)

Table S4 Functionally enriched terms for the up-regulated genes after TSA treatment. TermIDs as from GO (Gene Ontology); WP corresponds to WikiPathways, used with KEGG and REACTOME as database sources.
(DOCX)

Table S5 Functionally enriched terms for the down-regulated genes after TSA treatment. TermIDs as from GO (Gene Ontology); WP corresponds to WikiPathways, used with KEGG and REACTOME as database sources.
(DOCX)

Table S6 Functionally enriched terms including both up- and down-regulated genes after TSA treatment. TermIDs as from GO (Gene Ontology); WP corresponds to WikiPathways, used with KEGG and REACTOME as database sources.
(DOCX)

Table S7 Functionally enriched terms for the up-regulated genes after combined DAC+TSA treatment. TermIDs as from GO (Gene Ontology); WP corresponds to WikiPathways, used with KEGG and REACTOME as database sources.
(DOCX)

Table S8 Functionally enriched terms for the down-regulated genes after combined DAC+TSA treatment. TermIDs as from GO (Gene Ontology); WP corresponds to WikiPathways, used with KEGG and REACTOME as database sources.
(DOCX)
