# Supplementary figures and images for: Separate and Combined Effects of DNMT and HDAC Inhibitors in Treating Human Multi-Drug Resistant Osteosarcoma HosDXR150 Cell Line
Source: PLoS One. 2014 Apr 22;9(4):e95596. doi: 10.1371/journal.pone.0095596 (PMC3995708; doi:10.1371/journal.pone.0095596)

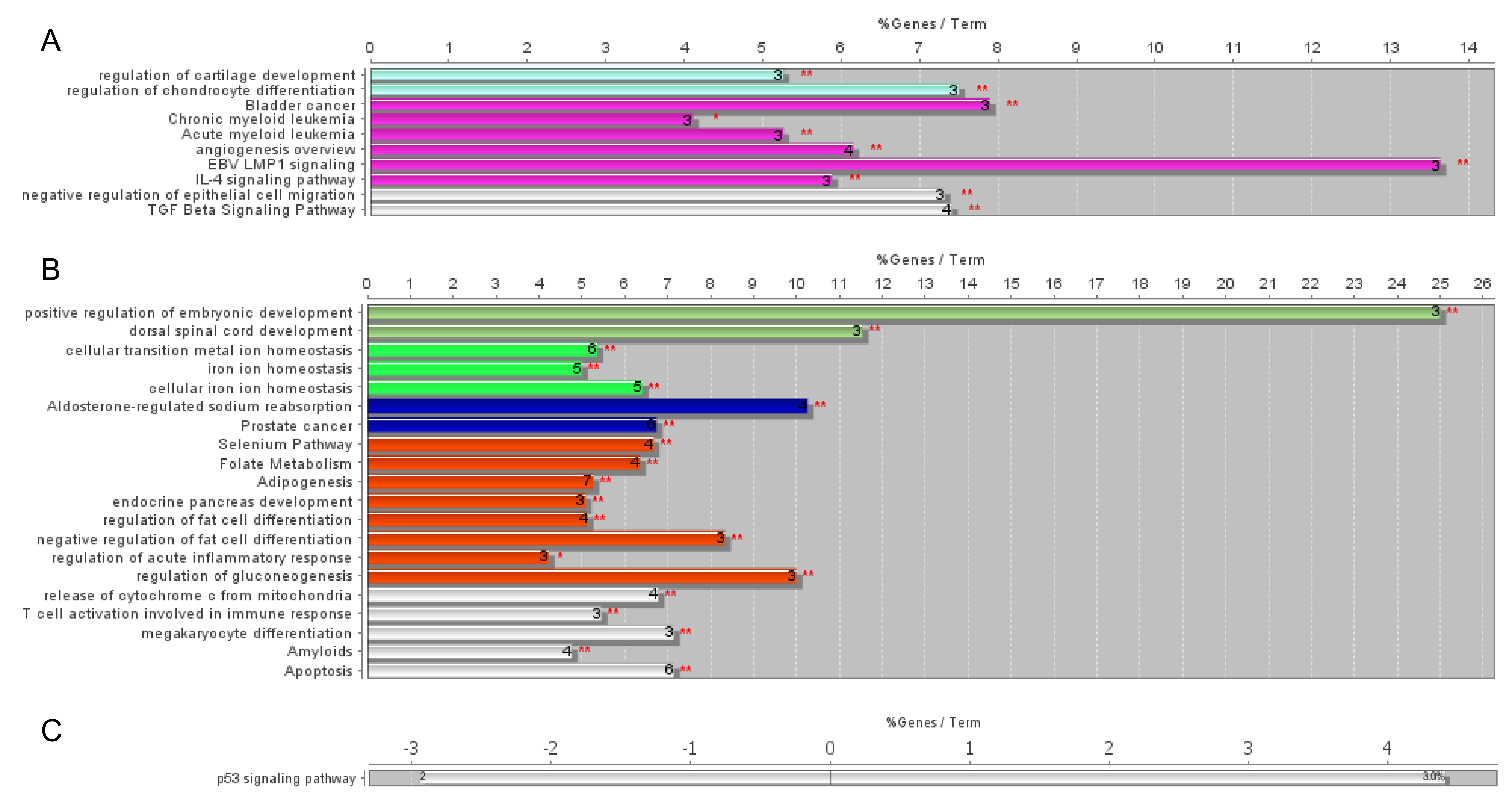

Supplement: Figure S1 — Functionally enriched terms for the up- and down-regulated genes after DAC treatment. (a) Pathways and GO terms enriched in up-regulated genes after DAC treatment; (b) Pathways and GO terms enriched in down-regulated genes after DAC treatment; (c) Pathways and GO terms enriched in both up- and down-regulated genes. ClueGO has provided the functional clusters; the number of associated genes for each cluster are reported within the bars. (TIF) [file pone.0095596.s001.tif]

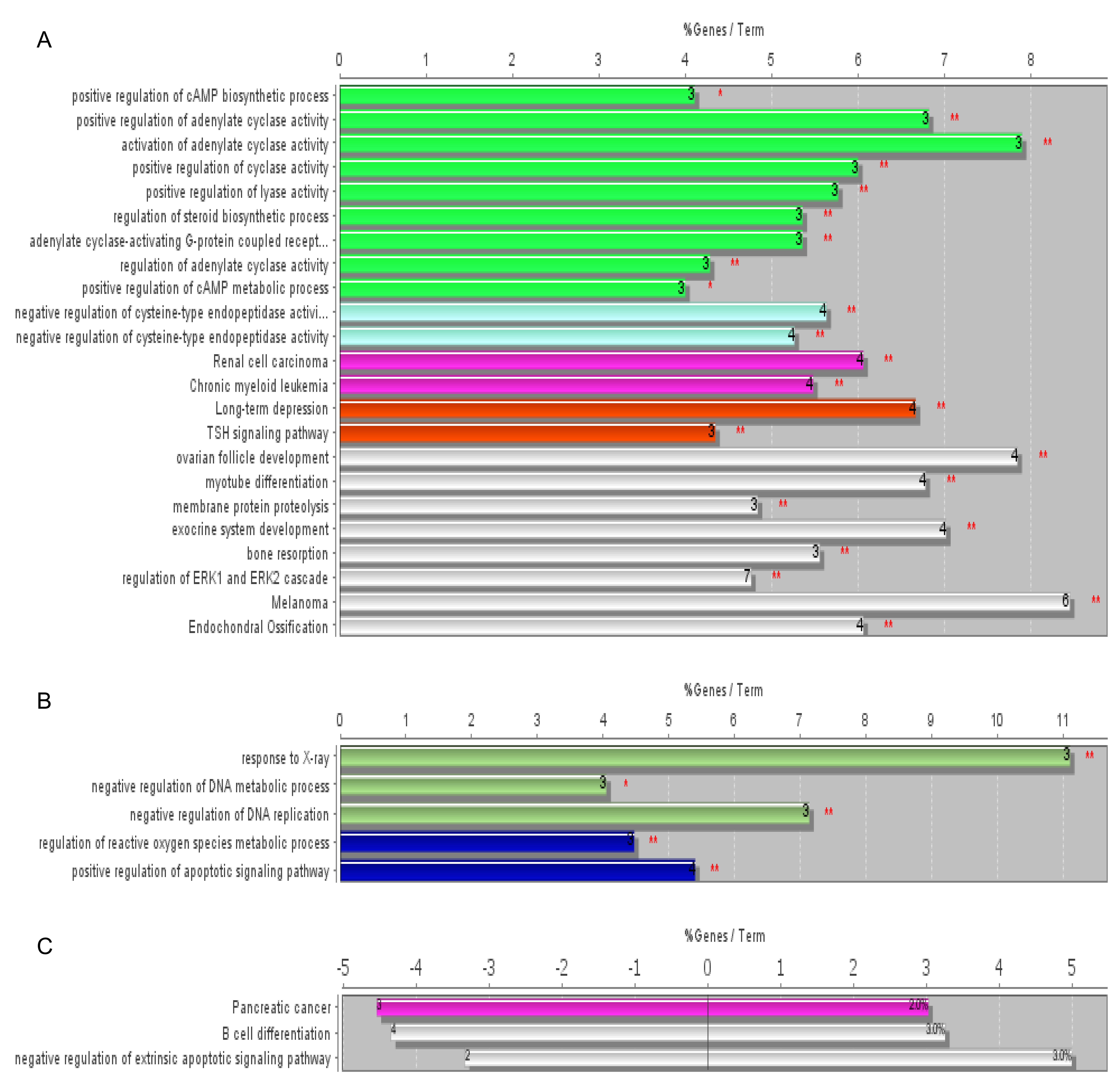

Supplement: Figure S2 — Functionally enriched terms after TSA treatment. (a) Pathways and GO terms enriched in up-regulated genes after TSA; (b) Pathways and GO terms enriched in down-regulated genes after TSA; (c) Pathways and GO terms enriched in both up- and down-regulated genes. ClueGO has provided the functional clusters. The number of associated genes for each cluster are reported within the bars. (TIF) [file pone.0095596.s002.tif]

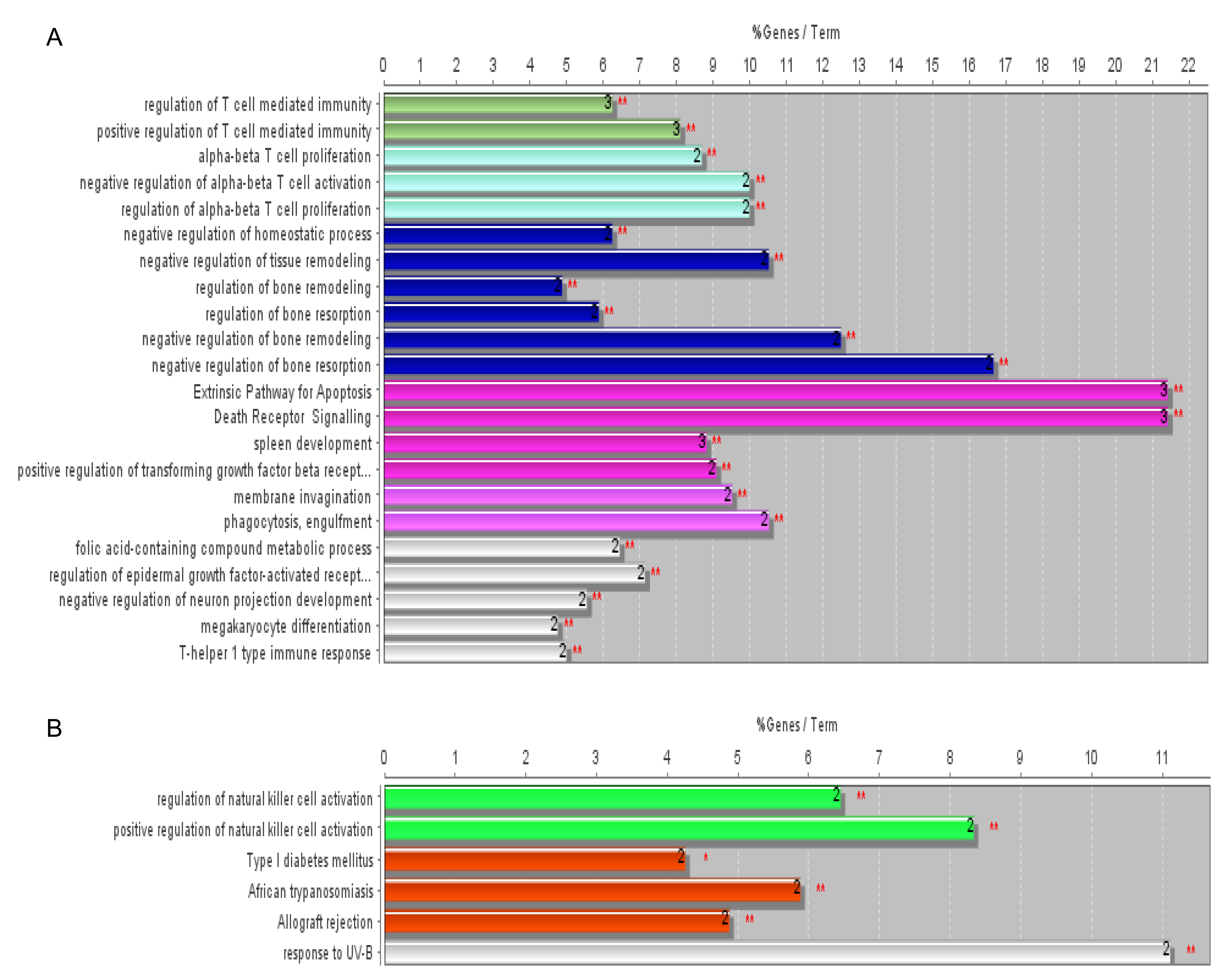

Supplement: Figure S3 — Functionally enriched terms after combined DAC+TSA treatment. (a) Pathways and GO terms enriched in up-regulated genes after DAC+TSA; (b) Pathways and GO terms enriched in down-regulated genes after DAC+TSA. ClueGO has provided the functional clusters. The number of associated genes for each cluster are reported within the bars. (TIF) [file pone.0095596.s003.tif]
