# Supplementary material for: Separate and Combined Effects of DNMT and HDAC Inhibitors in Treating Human Multi-Drug Resistant Osteosarcoma HosDXR150 Cell Line
Source: PLoS One. 2014 Apr 22;9(4):e95596. doi: 10.1371/journal.pone.0095596 (PMC3995708; doi:10.1371/journal.pone.0095596)
Supplement: Table S1 — Functionally enriched terms for the up-regulated genes after DAC treatment. TermIDs as from GO (Gene Ontology); WP corresponds to WikiPathways, used with KEGG and REACTOME as database sources. (DOCX) [file pone.0095596.s004.docx]

**Table S1**

| Term | TermID | Corrected p-value | Associated Genes |
| --- | --- | --- | --- |
| Adipogenesis | WP:236 | 0.000428803 | FOXO1, IGF1, IL6, IL6ST, INS, RBL2, WNT1 |
| positive regulation of embryonic development | GO:0040019 | 0.000535755 | LHX1, NR2C2, WNT1 |
| Aldosterone-regulated sodium reabsorption | KEGG:04960 | 0.000680062 | IGF1, INS, MAPK1, SFN |
| cellular iron ion homeostasis | GO:0006879 | 0.000834421 | ATP6V1B1, CP, MFI2, MYC, PRMT1 |
| Apoptosis | WP:254 | 0.000835037 | BNIP3L, IGF1, MYC, RELA, TNFRSF1B, TP73 |
| Selenium Pathway | WP:15 | 0.001799368 | IL6, INS, RELA, XDH |
| release of cytochrome c from mitochondria | GO:0001836 | 0.00181839 | IL6, MYC, SFN, TP73 |
| dorsal spinal cord development | GO:0021516 | 0.001964131 | LHX1, PBX3, WNT1 |
| Folate Metabolism | WP:176 | 0.002018507 | IL6, INS, RELA, SLC19A1 |
| regulation of gluconeogenesis | GO:0006111 | 0.002121088 | FOXO1, IL6, INS |
| regulation of fat cell differentiation | GO:0045598 | 0.003519736 | FOXO1, IL6, INS, WNT1 |
| Amyloids | REACTOME:75925 | 0.003981304 | HIST1H4I, INS, LYZ, MFGE8 |
| megakaryocyte differentiation | GO:0030219 | 0.003995663 | HIST1H4I, PRMT1, PSG1 |
| T cell activation involved in immune response | GO:0002286 | 0.007889313 | IL6, MYB, TNFSF18 |
| endocrine pancreas development | GO:0031018 | 0.008895339 | FOXO1, IL6, INS |
| regulation of acute inflammatory response | GO:0002673 | 0.01421018 | IL6, IL6ST, INS |

**Table S1.** **Functionally enriched terms for the up-regulated genes after DAC treatment**. TermIDs as from GO (Gene Ontology); WP corresponds to WikiPathways, used with KEGG and REACTOME as database sources
