# Supplementary material for: Separate and Combined Effects of DNMT and HDAC Inhibitors in Treating Human Multi-Drug Resistant Osteosarcoma HosDXR150 Cell Line
Source: PLoS One. 2014 Apr 22;9(4):e95596. doi: 10.1371/journal.pone.0095596 (PMC3995708; doi:10.1371/journal.pone.0095596)
Supplement: Table S2 — Functionally enriched terms for the down-regulated genes after DAC treatment. TermIDs as from GO (Gene Ontology); WP corresponds to WikiPathways, used with KEGG and REACTOME as database sources. (DOCX) [file pone.0095596.s005.docx]

**Table S2**

| Term | TermID | Corrected p-value | Associated Genes |
| --- | --- | --- | --- |
| EBV LMP1 signaling | WP:262 | 0.001583304 | IFNB1, MAPK1, RELA |
| TGF Beta Signaling Pathway | WP:560 | 0.001687583 | ENG, SMAD5, TFE3, WNT1 |
| angiogenesis overview | WP:1993 | 0.002127782 | DAG1, MAPK1, MMP2, TIMP4 |
| Bladder cancer | KEGG:05219 | 0.003596069 | MAPK1, MMP2, MYC |
| negative regulation of epithelial cell migration | GO:0010633 | 0.003891368 | ACVRL1, CXCL13, MCC |
| regulation of chondrocyte differentiation | GO:0032330 | 0.003970325 | ACVRL1, RELA, SAFB |
| negative regulation of endothelial cell proliferation | GO:0001937 | 0.0061317 | ACVRL1, ENG, XDH |
| IL-4 signaling pathway | WP:395 | 0.006646647 | FES, MAPK1, RELA |
| Acute & Chronic myeloid leukemia | KEGG:05221 | 0.008387515 | MAPK1, MYC, RELA |
| regulation of cartilage development | GO:0061035 | 0.008387515 | ACVRL1, RELA, SAFB |

**Table S2.** **Functionally enriched terms for the down-regulated genes after DAC treatment.** TermIDs as from GO (Gene Ontology); WP corresponds to WikiPathways, used with KEGG and REACTOME as database sources.
