# Supplementary material for: Separate and Combined Effects of DNMT and HDAC Inhibitors in Treating Human Multi-Drug Resistant Osteosarcoma HosDXR150 Cell Line
Source: PLoS One. 2014 Apr 22;9(4):e95596. doi: 10.1371/journal.pone.0095596 (PMC3995708; doi:10.1371/journal.pone.0095596)
Supplement: Table S3 — Functionally enriched terms including both up- and down-regulated genes after DAC treatment. TermIDs as from GO (Gene Ontology); WP corresponds to WikiPathways, used with KEGG and REACTOME as database sources. (DOCX) [file pone.0095596.s006.docx]

| Term | TermID | Corrected p-value | Associated Genes |
| --- | --- | --- | --- |
| p53 signaling pathway | KEGG:04115 | 0.000697129 | BAI1, EI24, IGF1, SFN, TP73 |

**Table S3**

**Table S3.** **Functionally enriched terms including both up- and down-regulated genes after DAC treatment**. TermIDs as from GO (Gene Ontology); WP corresponds to WikiPathways, used with KEGG and REACTOME as database sources.
