# Supplementary material for: Separate and Combined Effects of DNMT and HDAC Inhibitors in Treating Human Multi-Drug Resistant Osteosarcoma HosDXR150 Cell Line
Source: PLoS One. 2014 Apr 22;9(4):e95596. doi: 10.1371/journal.pone.0095596 (PMC3995708; doi:10.1371/journal.pone.0095596)
Supplement: Table S4 — Functionally enriched terms for the up-regulated genes after TSA treatment. TermIDs as from GO (Gene Ontology); WP corresponds to WikiPathways, used with KEGG and REACTOME as database sources. (DOCX) [file pone.0095596.s007.docx]

| Term | TermID | Corrected p-value | Associated Genes |
| --- | --- | --- | --- |
| response to X-ray | GO:0010165 | 0.001574959 | BLM, ERCC6, TP53 |
| positive regulation of apoptotic signaling pathway | GO:2001235 | 0.002064234 | AGFG1, LCK, TP53, WWOX |
| negative regulation of DNA replication & metabolic process | GO:0008156 | 0.003408117 | BLM, RAD17, TP53 |
| B cell receptor signaling pathway | GO:0050853 | 0.003620938 | CD38, LCK, NCKAP1L |

**Table S4.**

**Table S4. Functionally enriched terms for the up-regulated genes after TSA treatment.** TermIDs as from GO (Gene Ontology); WP corresponds to WikiPathways, used with KEGG and REACTOME as database sources.
