# Supplementary material for: Separate and Combined Effects of DNMT and HDAC Inhibitors in Treating Human Multi-Drug Resistant Osteosarcoma HosDXR150 Cell Line
Source: PLoS One. 2014 Apr 22;9(4):e95596. doi: 10.1371/journal.pone.0095596 (PMC3995708; doi:10.1371/journal.pone.0095596)
Supplement: Table S5 — Functionally enriched terms for the down-regulated genes after TSA treatment. TermIDs as from GO (Gene Ontology); WP corresponds to WikiPathways, used with KEGG and REACTOME as database sources. (DOCX) [file pone.0095596.s008.docx]

**Table S5**

| Term | TermID | Corrected p-value | Associated Genes |
| --- | --- | --- | --- |
| Melanoma | KEGG:05218 | 5.88280803353884e-05 | BRAF, FGF12, FGF20, HGF, IGF1R, TP53 |
| regulation of ERK1 and ERK2 cascade | GO:0070372 | 0.000270818 | BRAF, FGF20, GPR183, IGFBP1, ROS1, ST5, WNK2 |
| ovarian follicle development | GO:0001541 | 0.00116159 | INHA, LHCGR, MSH4, VEGFA |
| Long-term depression | KEGG:04730 | 0.001450347 | BRAF, GNA13, IGF1R, PLCB3 |
| myotube differentiation | GO:0014902 | 0.001529819 | BCL9, CFLAR, IGFBP1, NEO1 |
| exocrine system development | GO:0035272 | 0.001531321 | BMP7, HGF, IGF1R, IGFBP1 |
| Endochondral Ossification | WP:474 | 0.001706588 | BMP7, CTSV, IGF1R, VEGFA |
| Renal cell carcinoma | KEGG:05211 | 0.001706588 | BRAF, HGF, TGFA, VEGFA |
| negative regulation of cysteine-type endopeptidase activity involved in apoptotic process | GO:0043154 | 0.00206157 | HGF, IGFBP1, RAG1, VEGFA |
| Chronic myeloid leukemia | KEGG:05220 | 0.002112438 | BRAF, CBLB, RELA, TP53 |
| activation of adenylate cyclase activity & biosynthetic process | GO:0007190 | 0.00270724 | ADRB2, CALCR, LHCGR |
| Negative regulation of bone resorption | GO:0045453 | 0.005661156 | ADRB2, CD38, VEGFA |
| adenylate cyclase-activating G-protein coupled receptor signaling pathway | GO:0007189 | 0.005987474 | ADRB2, CALCR, GNA13 |
| regulation of steroid biosynthetic process | GO:0050810 | 0.005987474 | ADRB2, IGF1R, LHCGR |
| membrane protein proteolysis | GO:0033619 | 0.00761535 | ADAM17, NAPSA, RELA |
| TSH signaling pathway | WP:2032 | 0.009419343 | BRAF, GNA13, IGF1R |

**Table S5.** **Functionally enriched terms for the down-regulated genes after TSA treatment.** TermIDs as from GO (Gene Ontology); WP corresponds to WikiPathways, used with KEGG and REACTOME as database sources.
