# Supplementary material for: Separate and Combined Effects of DNMT and HDAC Inhibitors in Treating Human Multi-Drug Resistant Osteosarcoma HosDXR150 Cell Line
Source: PLoS One. 2014 Apr 22;9(4):e95596. doi: 10.1371/journal.pone.0095596 (PMC3995708; doi:10.1371/journal.pone.0095596)
Supplement: Table S6 — Functionally enriched terms including both up- and down-regulated genes after TSA treatment. TermIDs as from GO (Gene Ontology); WP corresponds to WikiPathways, used with KEGG and REACTOME as database sources. (DOCX) [file pone.0095596.s009.docx]

**Table S6**

| Term | TermID | Corrected p-value | Associated Genes |
| --- | --- | --- | --- |
| B cell differentiation | GO:0030183 | 3.65112603857156e-05 | ADAM17, GPR183, INHA, MALT1, NCKAP1L, RAG1, TP53 |
| negative regulation of extrinsic apoptotic signaling pathway | GO:2001237 | 0.000208611 | AGFG1, CFLAR, HTT, IGFBP1, RELA |
| Pancreatic cancer | KEGG:05212 | 0.000265477 | BRAF, RELA, TGFA, TP53, VEGFA |

**Table S6.** **Functionally enriched terms including both up- and down-regulated genes after TSA treatment**. TermIDs as from GO (Gene Ontology); WP corresponds to WikiPathways, used with KEGG and REACTOME as database sources.
