# Supplementary material for: Separate and Combined Effects of DNMT and HDAC Inhibitors in Treating Human Multi-Drug Resistant Osteosarcoma HosDXR150 Cell Line
Source: PLoS One. 2014 Apr 22;9(4):e95596. doi: 10.1371/journal.pone.0095596 (PMC3995708; doi:10.1371/journal.pone.0095596)
Supplement: Table S7 — Functionally enriched terms for the up-regulated genes after combined DAC+TSA treatment. TermIDs as from GO (Gene Ontology); WP corresponds to WikiPathways, used with KEGG and REACTOME as database sources. (DOCX) [file pone.0095596.s010.docx]

**Table S7**

| Term | TermID | Corrected p-value | Associated Genes |
| --- | --- | --- | --- |
| Positive regulation natural killer cell activation | GO: 0032816 | 0.005057671 | AXL, IL12A |
| response to UV-B | GO:0010224 | 0.005275466 | IL12A, XPC |
| African trypanosomiasis | KEGG:05143 | 0.007692111 | FAS, IL12A |
| Allograft rejection | KEGG:05330 | 0.00941264 | FAS, IL12A |
| Type I diabetes mellitus | KEGG:04940 | 0.011134708 | FAS, IL12A |

**Table S7.** **Functionally enriched terms for the up-regulated genes after combined DAC+TSA treatment**. TermIDs as from GO (Gene Ontology); WP corresponds to WikiPathways, used with KEGG and REACTOME as database sources.
