# Supplementary material for: Separate and Combined Effects of DNMT and HDAC Inhibitors in Treating Human Multi-Drug Resistant Osteosarcoma HosDXR150 Cell Line
Source: PLoS One. 2014 Apr 22;9(4):e95596. doi: 10.1371/journal.pone.0095596 (PMC3995708; doi:10.1371/journal.pone.0095596)
Supplement: Table S8 — Functionally enriched terms for the down-regulated genes after combined DAC+TSA treatment. TermIDs as from GO (Gene Ontology); WP corresponds to WikiPathways, used with KEGG and REACTOME as database sources. (DOCX) [file pone.0095596.s011.docx]

**Table S8**

| Term | TermID | Corrected p-value | Associated Genes |
| --- | --- | --- | --- |
| Extrinsic Pathway for Apoptosis | REACTOME:1059 | 0.000290735 | ADAM17, CASP10, FAS |
| Death Receptor Signaling | REACTOME:1619 | 0.000290735 | ADAM17, CASP10, FAS |
| positive regulation of T cell mediated immunity | GO:0002711 | 0.001956993 | HFE, IL12A, TNFSF4 |
| spleen development | GO:0048536 | 0.002277151 | ADAM17, CDKN2B, FAS |
| negative regulation of bone resorption | GO:0045779 | 0.003227988 | CD38, VEGFA |
| negative regulation of bone remodeling | GO:0046851 | 0.004848343 | CD38, VEGFA |
| positive regulation of TGF-beta receptor signaling pathway | GO:0030511 | 0.005024553 | ADAM17, CDKN2B |
| alpha-beta T cell proliferation | GO:0046633 | 0.005033507 | CBLB, TNFSF4 |
| membrane invagination | GO:0010324 | 0.005035521 | BIN2, FCGR1A |
| phagocytosis, engulfment | GO:0006911 | 0.00514785 | BIN2, FCGR1A |
| negative regulation of tissue remodeling | GO:0034104 | 0.00514785 | CD38, VEGFA |
| regulation of epidermal growth factor-activated receptor activity | GO:0007176 | 0.006376239 | ADAM17, CBLC |
| negative regulation of homeostatic process | GO:0032845 | 0.007257418 | CD38, VEGFA |
| folic acid-containing compound metabolic process | GO:0006760 | 0.007273223 | FOLH1, FOLR1 |
| negative regulation of neuron projection development | GO:0010977 | 0.00812291 | CBFA2T2, STMN2 |
| megakaryocyte differentiation | GO:0030219 | 0.009392235 | CDKN2B, PSG1 |
| T-helper 1 type immune response | GO:0042088 | 0.009445204 | TNFSF4, VEGFA |

**Table S8.** **Functionally enriched terms for the down-regulated genes after combined DAC+TSA treatment.** TermIDs as from GO (Gene Ontology); WP corresponds to WikiPathways, used with KEGG and REACTOME as database sources.
